# Knockdown of SFRS9 Inhibits Progression of Colorectal Cancer Through Triggering Ferroptosis Mediated by GPX4 Reduction

**DOI:** 10.3389/fonc.2021.683589

**Published:** 2021-07-16

**Authors:** Rui Wang, Rui Xing, Qi Su, Hongzhuan Yin, Di Wu, Chi Lv, Zhaopeng Yan

**Affiliations:** ^1^ Department of Critical Care Medicine, Shengjing Hospital of China Medical University, Shenyang, China; ^2^ Department of Oncology, Shengjing Hospital of China Medical University, Shenyang, China; ^3^ Department of General Surgery, Shengjing Hospital of China Medical University, Shenyang, China

**Keywords:** colorectal cancer, progression, serine and arginine rich splicing factor 9, glutathione peroxidase 4, ferroptosis

## Abstract

Ferroptosis, a newly discovered form of programmed cell death characterized by lipid peroxidation, crafts a new perspective on cancer treatment. Serine and arginine rich splicing factor 9 (SFRS9) is frequently described as a proto-oncogene in cervical and bladder cancer. However, the role of SFRS9 in colorectal cancer (CRC) and whether SFRS9 exerts its function associated with ferroptosis is largely unknown. Herein, we found that the expression of SFRS9 mRNA and protein in the CRC tissues was obviously higher than that in the paracancerous tissues. Function assays revealed that SFRS9 overexpression (SFRS9-OE) significantly promoted cell viability, cell cycle progression and colony formation of CRC cells. While SFRS9 knockdown by shRNAs transfection inhibited these progressions. Furthermore, cell death and lipid peroxidation induced by ferroptosis inducers erastin and sorafenib were suppressed by SFRS9-OE. Bioinformatics analysis indicated that SFRS9 can bind to peroxidase 4 (GPX4) mRNA which is a central regulator of ferroptosis. Western blot showed that GPX4 protein expression was clearly elevated upon SFRS9-OE, while it was decreased in SFRS9-inhibited CRC cells. RNA immunoprecipitation experiment was carried out in HCT116 cells to confirm the binding of SFRS9 and GPX4 mRNA specifically. SiGPX4 transfection reversed the inhibitory effects of SFRS9-OE on the erastin and sorafenib-induced ferroptosis. Consistent with our *in vitro* observations, SFRS9 promoted the growth of tumors while SFRS9 knockdown significantly inhibited tumor growth in nude mice. In conclusion, SFRS9 represents an obstructive factor to ferroptosis by upregulating GPX4 protein expression, and knocking down SFRS9 might be an effective treatment for CRC.

## Introduction

Colorectal cancer (CRC) is one of the most deadly cancers and it causes almost 700,000 deaths every year (1). Environmental and genetic factors are considered to play a major role in the pathogenesis of CRC. Also, westernization of dietary habits, low physical exercise and obesity are thought to be associated with an increased incidence of CRC as the world becomes richer (1). In a nutshell, CRC is a serious disease that poses a significant threat to human health. New therapeutic strategies such as laparoscopic surgery for primary disease, more-aggressive resection of metastatic disease, radiotherapy for rectal cancer and neoadjuvant chemotherapies have been continuously developed, providing more treatment options for CRC patients (2). However, the effectiveness of these treatments is limited, resulting in unsatisfactory outcomes, cure rates and long-term survival. Thus, exploring new therapeutic targets to prevent the tumorigenesis of CRC is urgently needed.

Ferroptosis, a newly discovered type of programmed cell death, is distinct from necrosis, autophagy, and apoptosis (3). Ferroptosis is driven by the suppression of lipid repair enzyme glutathione peroxidase 4 (GPX4), and subsequent accumulation of iron-dependent lipid reactive oxygen species (ROS). Accumulated evidences suggest that ferroptosis occurs under physiological conditions and in various diseases such as brain and cardiovascular disease and cancers (4–6). With regard to cancer treatments, one of the most difficult challenges is to effectively kill cancer cells while protecting healthy cells. Cancer cells need more iron to enable growth than normal cells and their dependence on iron makes them more susceptible to ferroptosis (7). Ferroptosis crafts a new perspective on cancer treatments. Studies have reported ferroptosis as an anti-tumor factor in non-small cell lung carcinoma (8), liver cancer (9) and renal cancer (10). Furthermore, ferroptosis activated by small molecules or regulated by some genes could effectively suppress the progression of CRC (11, 12). However, the molecular mechanism of ferroptosis occurrence in CRC remains unclear.

SFRS9 (serine/arginine-rich splicing factor 9) is a protein encoded by a gene which is a member of the serine/arginine (SR)-rich family of pre-mRNA splicing factors. Each of these factors contains an RNA recognition motif (RRM) that binds RNA and an RS domain that binds protein. SR proteins are involved in splicing, mRNA transport, formation of the translational initiation complex and other processes of protein regulation (13). Emerging evidence indicates that SFRS9 is frequently overexpressed in various tumor types and behaves as a proto-oncogene (14–16). However, the role of SFRS9 in CRC has not yet been examined. Bioinformatics analysis revealedSFRS9 can bind to GPX4 mRNA which is a key factor in ferroptosis, and its expression in colorectal cancer is significantly positively correlated with GPX4. Therefore, we speculated that SFRS9 can interact with GPX4 mRNA and it is involved in the regulation of ferroptosis in CRC. The present study was performed to validate our speculation.

## Materials and Methods

### Ethics

The CRC samples used were from the Biobank, Shengjing Hospital of China Medical University. The study was approved by the Ethics Committee of the Shengjing Hospital of China Medical University.

### Cell Culture

Human CRC cell lines SW480, SW620, HT29, Colo205, Caco-2 and HCT116 were purchased from Shanghai Zhong Qiao Xin Zhou Biotechnology Co.,Ltd (Shanghai, China). Caco-2, SW620 and Colo205 cells were cultured in RPMI-1640 medium supplemented with 10% fetal bovine serum (FBS), HCT116 cells were cultured in DMEM medium supplemented with 10% FBS, HT29 were cultured in McCoy’s 5A medium supplemented with 10% FBS, SW480 were cultured in Leibovitzs L-15 medium supplemented with 10% FBS. All types of culture mediums were purchased from Shanghai Zhong Qiao Xin Zhou Biotechnology Co., Ltd. All CRC cells were maintained in an incubator under the condition of 5% CO2 at 37°C.

### Cell Transfection

The SFRS9-shRNA1 (5’-GATCCGGAAGGATCACATGCGAGAATTCAAGAGATTCTCGCATGTGATCCTTCTTTTTA-3’), SFRS9-shRNA2 (5’-GATCCGGCTGATGTGCAGAAGGATGTTCAAGAGACATCCTTCTGCACATCAGCTTTTTA-3’), and small interfering RNA (siRNA) oligonucleotides with sequence targeting GPX4 (5’-CAGGGAGUAACGAAGAGAU-3’) were purchased from Genepharma Co., Ltd (Shanghai, China). To overexpress SFRS9, the SFRS9 coding sequence was inserted into the pcDNA3.1 vector (pcDNA3.1-SFRS9, SFRS9-OE vector). SFRS9-OE vector, GPX4 siRNA, the SFRS9 shRNAs (SFRS9-shRNA1 and SFRS9-shRNA2), or their negative control (NC) were transfected into cells using Lipofectamine™ 2000 (Invitrogen, Carlsbad, CA, USA).

### Cell Treatment

After transfection with SFRS9 shRNAs or NC shRNA for 24 h, transfected CRC cells were treated with ferrostatin-1 (0.5 μM) or ZVAD-FMK (10 μM) for 24 h as needed.

After transfection with SFRS9-OE vector or empty vector for 24 h, or co-transfection with SFRS9-OE vector and SFRS9 shRNA, CRC cells were treated with erastin (20 μM) and sorafenib (10 μM) for 24 h as needed.

### CCK8 Assay

The CCK-8 assay was carried out to detect cell viability. To explore the effects of SFRS9 on cell viability, CRC cells were incubated in 96-well plates with a density of 4×10^3^ cells/well and transfected with SFRS9 shRNAs or SFRS9-OE vector, then allowed to grow for 0, 12, 24, 48, 72 and 96 h. To explore the role of SFRS9 in ferroptosis of CRC cells, ferroptosis inhibitor (Ferrostatin-1, 0.5 μM) or inducers (Erastin, 20 μM; sorafenib, 10 μM) were added for 24 h after transfection. The apoptosis inhibitor ZVAD-FMK (10 μM) were added for 24 h after shRNAs transfection to evaluate whether SFRS9 was involved in the apoptosis of CRC cells. At different time points, 10 μl CCK-8 solution (Beyotime Biotechnology, Shanghai, China) was added to each well. After 1 h incubation, optical density values were measured at 450 nm with a microplate reader (BioTek, Winooski, Vermont, USA).

### Lipid Peroxidation Assay

C11-BODIPY dye obtained from ThermoFisher Scientific (Grand Island, NY, USA) was used to detect lipid peroxidation in CRC cells according to the manufacturer’s instructions.

### Quantitative Real-Time PCR Analysis

Total RNA was extracted by Tripure reagent (BioTeke, Beijing, China), followed by reverse transcription with Super M-MLV reverse transcriptase (BioTeke). SYBR-Green PCR Master Mix (BioTeke) was obtained for Real-time PCR. The specific primers were as follows: SFRS9, sense: 5’-CCCTGCGTAAACTGGATG-3’, anti-sense, 5’- ACCGAGACCGTGAGTAGCC-3’; β-actin sense: 5’- GGCACCCAGCACAATGAA-3’, anti-sense: 5’- TAGAAGCATTTGCGGTGG-3’. β-actin was used as an internal control.

### Western Blot Assay

Protein expressions were studied using western blot assay. Firstly, CRC cells or tumor tissues were lysed using Western and IP cell lysate (Beyotime Biotechnology), and the lysates were cleared by centrifugation at 10,000g for 5 min. After resolving on 12% SDS-PAGE gel, the proteins were transferred electrophoretically to polyvinylidene fluoride (PVDF) membranes and subsequently incubated with the primary antibody overnight. The various antibodies used for western blot were SFRS9 (1:1000) and GPX4 (1:1000) antibody which was purchased from ABclonal Technology (Wuhan, China). The units were normalized based on β-actin (1:1000, Santa Cruz Biotechnology, Dallas, Texas, USA). Thereafter, membranes were incubated with peroxidase-conjugated secondary antibody (1:5000, Beyotime Biotechnology) for 45 min at 37°C. The signals were visualized using Enhanced chemiluminescence (Beyotime) and the images were analyzed by Gel-pro analyzer software (Media Cybernetics, CA, USA).

### Colony Formation Assay

A total of 200 CRC cells were seeded in a 35-mm cell culture dish and cultured for 14 days after treatments. After 14 days, colonies were fixed with 4% paraformaldehyde for 20 min at room temperature and stained with Wright-Giemsa dye (KeyGen Biotech Co.,Ltd, NanJing, China) for 5 min. The samples were photographed, and visible colonies were counted.

### EdU Proliferation Assay

EdU (5-ethynyl-2′-deoxyuridine) proliferation assay was performed with an EdU detection kit (KeyGen Biotech Co.,Ltd) to measure cell proliferation. At 48 h after transfection, CRC cells were plated in 6-well plates at a density of 2 ×  10^5^ cells/well, then treated with 10 nM Edu for 12 h. Then cells were fixed and permeabilized with 0.5% TritonX-100 for 20 min. Hereafter, Click-iT was added and cells were incubated for 30 min in the dark. After washing with PBS for two times, cells were counterstaining with Hoechst 33342 dye for 15-30 min in the dark and observed using a fluorescence microscope (IX53, Olympus, Tokyo, Japan).

### Cell Cycle Assay

CRC cells were cultured to a confluence of 90% and seeded in 6-well plates (2×10^5^/well). At 24 h after transfection, cells were cultured in an incubator with 5% CO_2_ at 37°C for 48 h. Then cells were collected, centrifuged (310 g, 5 min) and fixed in 70% cold ethanol (4°C, 12 h). After fixation, cell cycle of CRC cells was evaluated using a commercial Cell Cycle Assay kit (KeyGen Biotech Co.,Ltd) and analyzed by flow cytometry (NovoCyte, ACEA Biosciences Inc., San Diego, California, USA).

### Immunohistochemistry (IHC) Staining

IHC staining for SFRS9 and Ki67 was performed. Tumor tissue sections were obtained from paraffin blocks and rehydrated, and then incubated overnight with the primary antibodies including anti-SFRS9 (1:100, ABclonal Technology, Wuhan, China) and anti-Ki67 (1:100, ABclonal Technology). The sections were then washed with PBS, and incubated with the appropriate HRP-conjugated secondary antibody (1:500 dilution, ThermoFisher Scientific, Waltham, MA, USA) at 37°C for 1 h, and visualized with DAB. The haematoxylin (Solarbio) was used to counterstain the sections. Finally, the sealed slides were photographed under a microscope (BX53, Olympus).

### Bioinformatics Analysis

The online bioinformatic program Starbase v3.0 (http://starbase.sysu.edu.cn/) predicted that SFRS9 can bind to GPX4 mRNA.

### RNA Immunoprecipitation Assay

The cell lysate was prepared for immunoprecipitation. Cell lysate was incubated with magnetic particles conjugated with anti-SFRS9 antibody (Abcam, Cambridge, UK) at 4°C overnight. Then SFRS9-associated RNAs were immunoprecipitated, restored and purified. The mRNA was analyzed by PCR and the precipitated proteins were analyzed by western blot assay.

### *In Vivo* Tumor Xenograft Experiments

Animal experiments were premeditated and executed in accordance with the ethical norms approved by Shengjing Hospital of China Medical University and the guide for the Care and Use of Laboratory Animals published by the National Institutes of Health.

Caco-2 cells were stably transfected with SFRS9-OE or empty vector. HCT116 cells were stably transfected with SFRS9-shRNA1 or NC-shRNA. Then the transfected CRC cells (2 × 10^5^cells/100µl) were injected into the right armpit of 6 week-old male athymic nude mice (Beijing Huafukang Bioscience Co., Inc). Tumor volumes were measured every four days. Twenty-five days after injection, mice were sacrificed, and the tumor weights were recorded.

### Statistical Analyses

All data are showed as means ± standard deviations (SD). Experimental data were analyzed using Student’s t test, one-way or two-way analysis of variance (ANOVA) followed by Tukey’s test to compare the differences between different groups. Graphpad prism software (Graphpad 8.0, Inc, San Diego, CA, USA) was used for the statistical analyses. Statistical significance was considered as p < 0.05.

## Results

### Detection of SFRS9 Expression in Paracancerous and CRC Tissues

**Figure 1:**
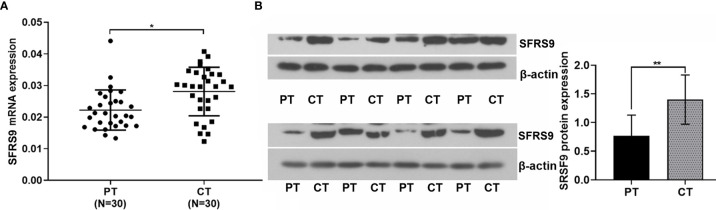
Expression of SFRS9 mRNA and protein in colon cancer tissues and paracancerous tissues. **(A)** Expression of SFRS9 mRNA level in different tissues (CT, CRC tissues; PT paracancerous tissues, N=30). **(B)** Expression of SFRS9 protein level in different tissues; N, 8). Values are mean ± SD. ^*^p < 0.05, ^**^p < 0.01.

Results showed that the SFRS9 mRNA expression in CRC tissues (CT) were higher than that in paracancerous tissues (PT) (N=30, **Figure 1A**). Similarly, the SFRS9 protein expression in CT was also significantly higher than that in the PT (N=8, **Figure 1B**).

### Selection of CRC Cells and the Efficiency of shRNAs Transfection

**Figure 2:**
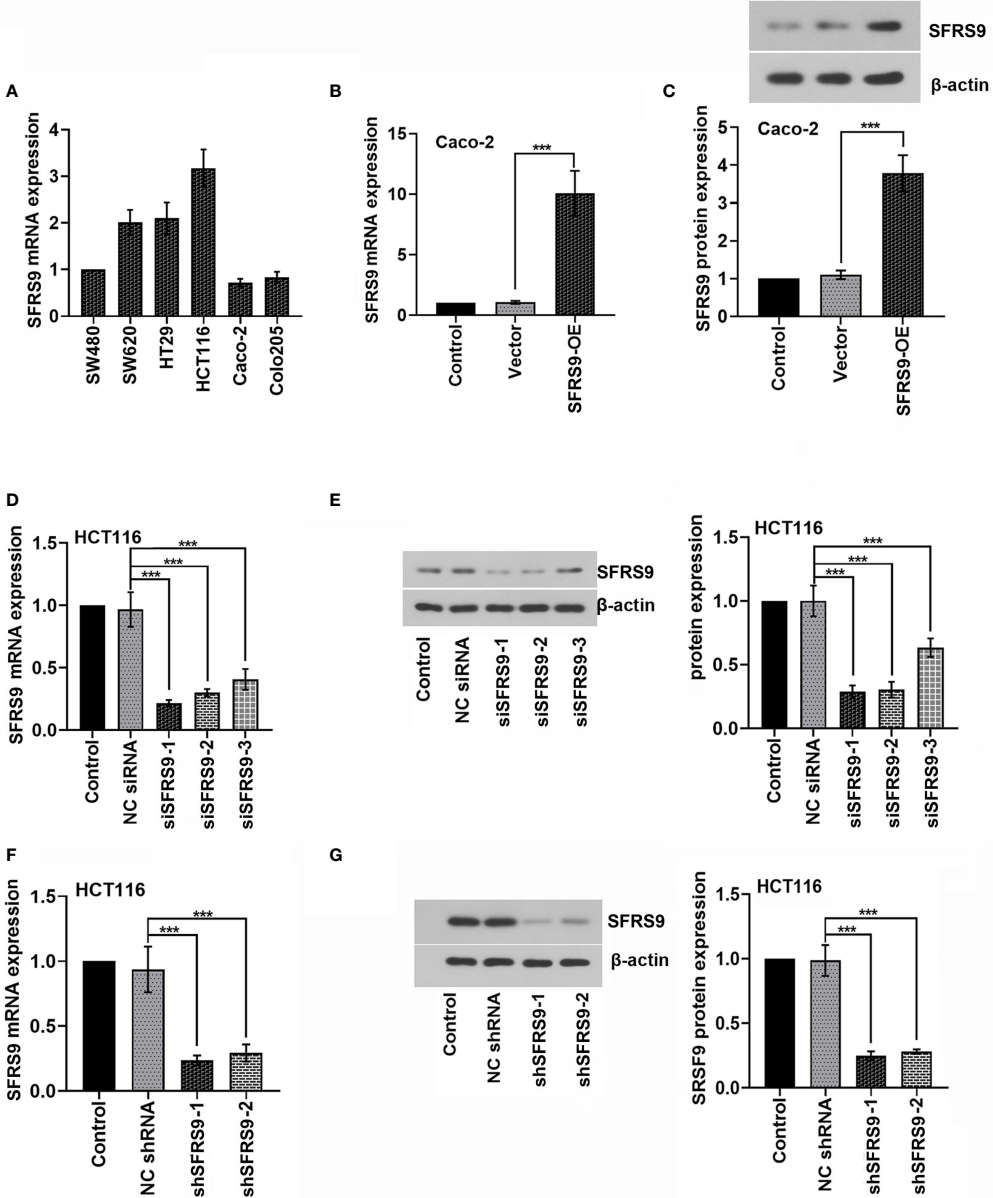
SFRS9 expression in colorectal (CRC) cells and inhibition of SFRS9 using RNAi. **(A)** qRT-PCR analysis of SFRS9 mRNA expression in the CRC lines. **(B, C)** mRNA and protein levels of SFRS9 in Caco-2 cells transfected with SFRS9 overexpression (SFRS9-OE) vector. **(D, E)** qRT-PCR and western blot analysis indicating the efficiency of siRNAs transfection in HCT116 cells. **(F, G)** shRNA specific for SFRS9 was designed. qRT-PCR and western blotting analysis indicated the efficiency of shRNAs transfection in HCT116 cells. Values are mean ± SD. ^***^p < 0.001.

To select suitable cell lines for the *in vitro* experiments, the level of SFRS9 mRNA in Caco-2, HCT116, SW620, HT29, SW480 and Colo205 CRC cells was determined. HCT116 CRC cells showed best SFRS9 mRNA expression, while Caco-2 cells showed relatively lower SFRS9 mRNA expression compared with other CRC cells (**Figure 2A**). We used HCT116 and Caco-2 cells for subsequent experiments. To illustrate the function of SFRS9 in CRC tumorigenesis, we firstly confirmed that SFRS9-OE vector transfection could significantly enhanced SFRS9 expression both at mRNA and protein levels in Caco-2 CRC cells (**Figures 2B, C**). Different siRNAs targeting SFRS9 could efficiently knockdown SFRS9 both at mRNA and protein expression in HCT116 cells, and siSFRS9-1 and siSFRS9-2 had the higher knockdown efficiency (**Figures 2D, E**). Next, shRNA-1 and shRNA-2 specific for SFRS9 were designed and were used in the subsequent experiments. Knockdown efficiency of shSFRS9-1 and shSFRS9-2 are shown in **Figures 2F, G**.

### Effects of SFRS9 on CRC Tumorigenesis *In Vitro*

**Figure 3:**
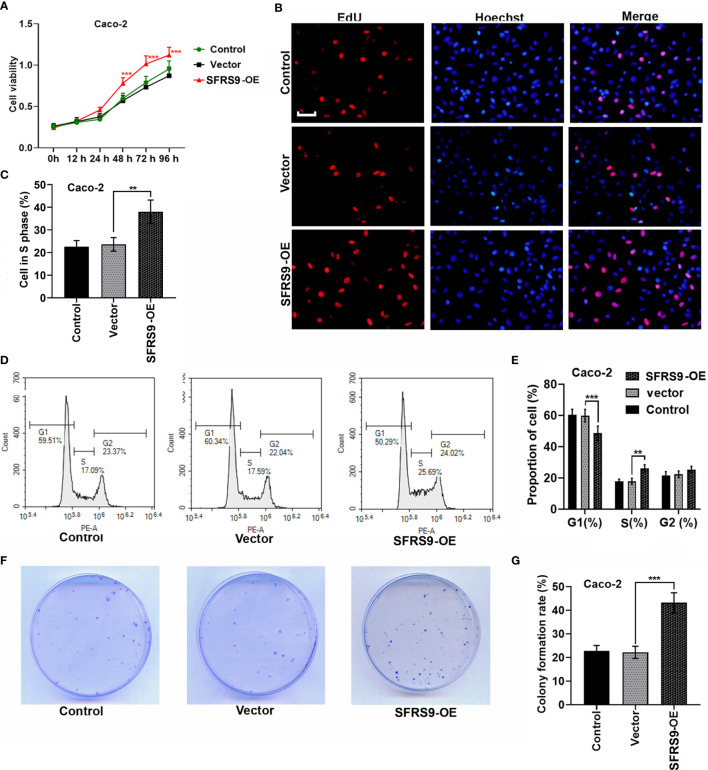
SFRS9 promoted the growth of CRC cells. **(A)** The viability of Caco-2 cells was measured using the CCK-8 assay (^***^p < 0.001 *vs.* Vector group). **(B, C)** Representative images and data analysis of EdU staining of Caco-2 cells transfected with SFRS9-OE vector, Scale bars: 50 μm. **(D, E)** Cell cycle analysis in Caco-2 cells transfected with SFRS9-OE vector. **(F, G)** Colony formation of Caco-2 cells transfected with SFRS9-OE vector was assessed. Values are mean ± SD. ^**^p < 0.01, ^***^p < 0.001.

To investigate the function of SFRS9 on CRC cell viability, we first overexpressed SFRS9 in Caco-2 cells and performed CCK8 assay. We found that SFRS9-OE significantly enhanced cell viability (**Figure 3A**). Then cell proliferation was assessed using an EdU assay, results showed that the percentage of Caco-2 cells in S phase significantly increased upon SFRS9-OE vector transfection (**Figures 3B, C**). The results of flow cytometry assay further revealed that the percentage of SFRS9-overexpressed Caco-2 cells in the G1 phase was lower than that of the Vector group, while SFRS9-OE obviously increased the percentage of cells in the S phase of the cell cycle (**Figures 3D, E**). In the colony formation assay, the number of colonies formed from SFRS9-overexpressed Caco-2 cells was significantly higher than that from vector-treated control (**Figures 3F, G**).

**Figure 4:**
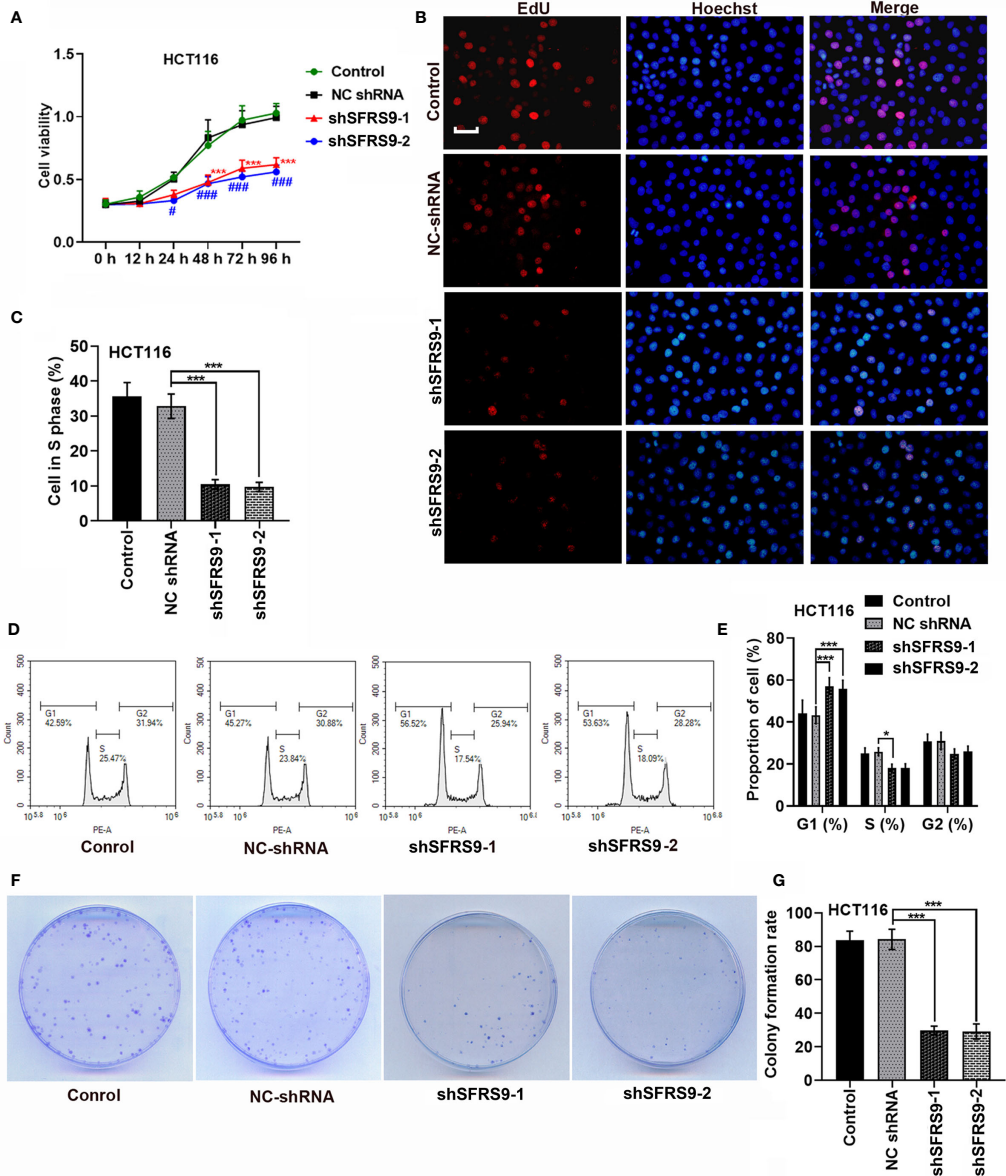
SFRS9 knockdown inhibited the growth of CRC cells. **(A)** The viability of HCT116 cells was measured using the CCK-8 assay (^***^p < 0.001 *vs.* NC shRNA group; ^#^p < 0.05, ^###^p < 0.001 vs. NC shRNA group). **(B, C)** Representative images and data analysis of EdU staining of HCT116 cells transfected with SFRS9 sh-RNAs, Scale bars: 50 μm. **(D, E)** Cell cycle analysis in HCT116 cells transfected with SFRS9 sh-RNAs. **(F, G)** Colony formation of HCT116 cells transfected with SFRS9 sh-RNAs was assessed. Values are mean ± SD. ^*^p < 0.05, ^***^p < 0.001.

Subsequently, shSFRS9-1 or shSFRS9-2 was transfected into HCT116 cells to further explore the role of SFRS9 in CRC tumorigenesis. CCK8 assay results showed shSFRS9s transfection significantly reduced cell viability (**Figure 4A**). Remarkably, SFRS9 knockdown decreased the percentage of Caco-2 cells in S phase (**Figures 4B, C**). Analysis of cell cycle showed that there was a statistically significant increase in the percentage of SFRS9-inhibited HCT116 cells in the G1 phase (**Figures 4D, E**). ShSFRS9-1 obviously reduced percentage of SFRS9-suppressed HCT116 cells in the S phase of the cell cycle, shSFRS9-2 also induced a decrease in the proportion of cells in the S phase although there was no statistical difference (**Figures 4D, E**). In the colony formation assay, the number of colonies formed from SFRS9-suppressed HCT116 cells was significantly lower than that from vector-treated control (**Figures 4F, G**).

### Effects of SFRS9 on Ferroptosis of CRC Cells

**Figure 5:**
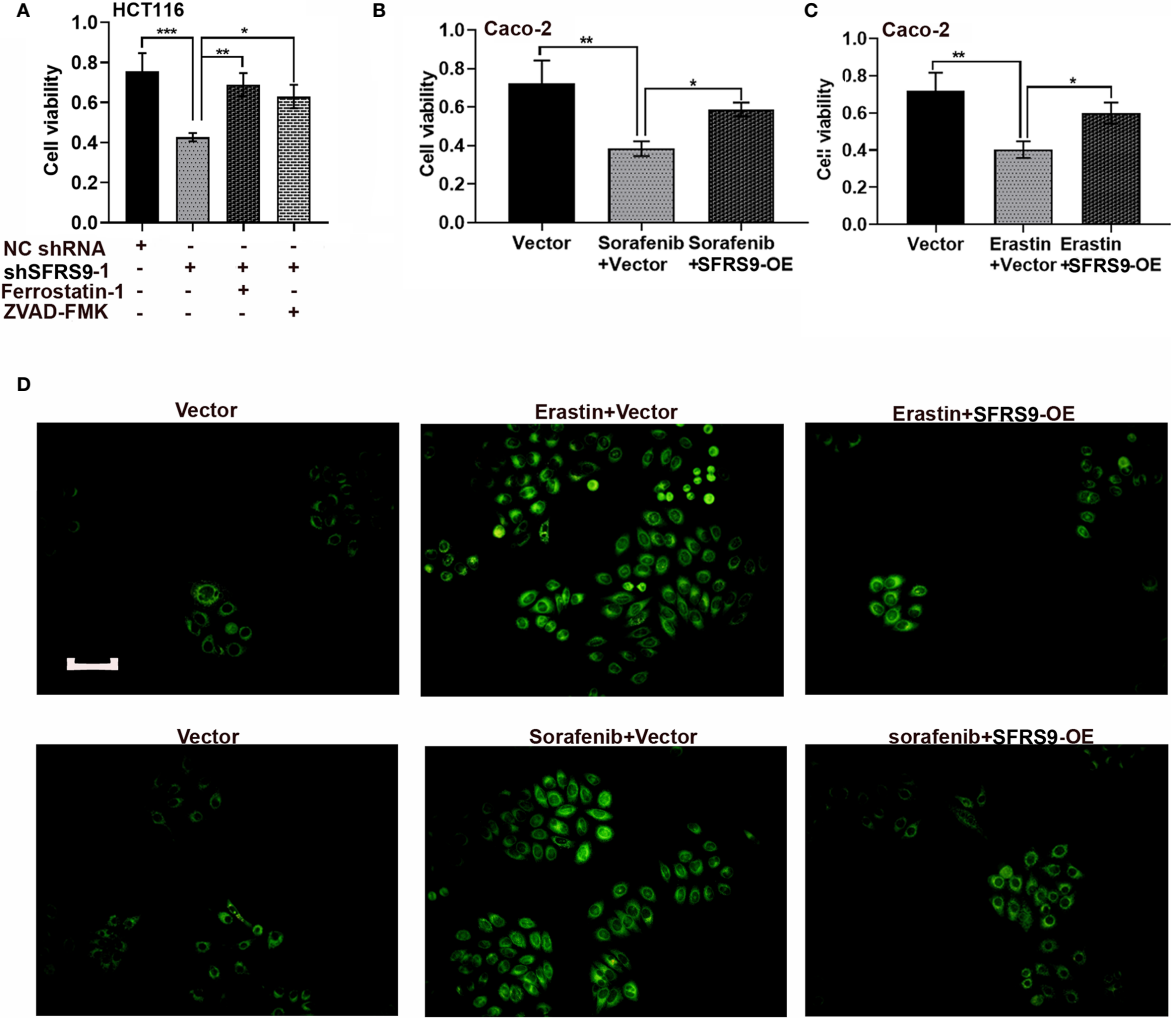
Effects of SFRS9 on ferroptosis in CRC cells. **(A)** The viability of HCT116 cells treated with SFRS9 shRNA with or without the indicated inhibitors was measured using the CCK-8 assay. **(B)** The viability of Caco-2 cells treated with SFRS9-OE vector with or without ferroptosis inducer sorafenib was measured using the CCK-8 assay. **(C)** The viability of Caco-2 cells treated with SFRS9 OE vector with or without ferroptosis inducer erastin was measured using the CCK-8 assay. **(D)** C11-BODIPY staining of Caco-2 cells following transfection with SFRS9-OE vector with or without ferroptosis inducer erastin or sorafenib. Scale bars: 100 μm. Values are mean ± SD. ^*^p < 0.05, ^**^p < 0.01, ^***^p < 0.001.

Ferroptosis is a new form of cell death. To assess whether ferroptosis mediated the tumor promotion of SFRS9 in CRC cells, we treated HCT116 cells with shSFRS9-1 in the absence or presence of ferroptosis inhibitor ferrostatin-1 and apoptosis inhibitor ZVAD-FMK. Our data showed both these inhibitors reversed SFRS9 knockdown induced viability inhibition in CRC cells (**Figure 5A**). Interestingly, ferroptosis inhibitor induced a slight increase in the prevention of SFRS9 knockdown-induced viability inhibition than the other inhibitor (**Figure 5A**). In **Figures 5B, C**, SFRS9-OE remarkably elevated cell viability which was inhibited by ferroptosis inducers erastin or sorafenib. Because the lipid peroxidation is a hallmark of ferroptosis, we next explored whether SFRS9 prevented the lipid peroxidation induced by ferroptosis inducers erastin and sorafenib. Lipid oxidation was measured by C11-BODIPY staining assay, a dye that detects lipid ROS. Results showed elevated SFRS9 expression inhibited the increase in oxidized lipids induced by ferroptosis inducers (**Figure 5D**).

### SFRS9 Could Bind to GPX4 mRNA and Regulate Its Protein Expression

**Figure 6:**
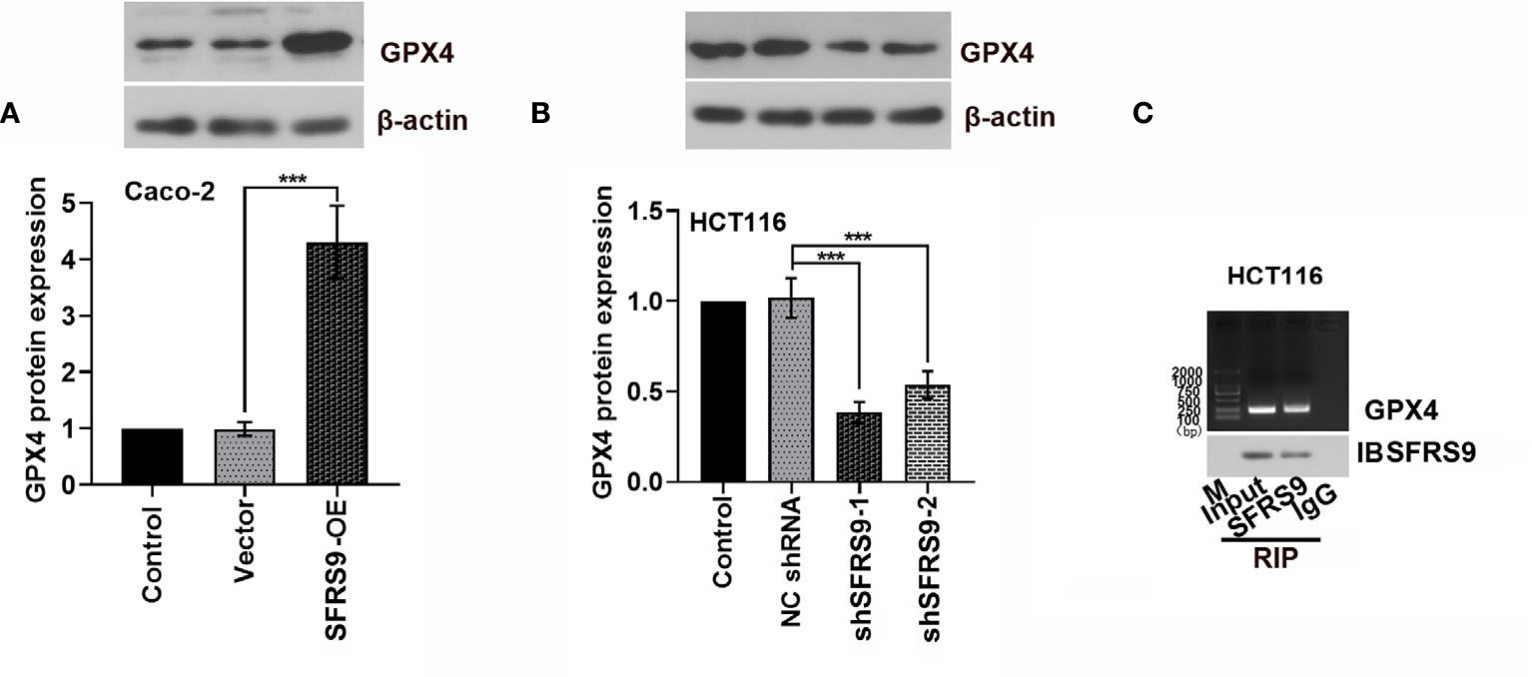
SFRS9 regulation of GPX4. **(A)** Protein levels of GPX4 in Caco-2 cells transfected with SFRS9 OE vector. **(B)** Protein levels of GPX4 in HCT116 cells transfected with SFRS9 shRNAs. **(C)** RNA immunoprecipitation assay was performed to evaluate the interaction between SFRS9 and GPX4. Values are mean ± SD. ^***^p < 0.001.

Bioinformatics analysis revealed that SFRS9 might bind to GPX4 mRNA. To test whether SFRS9 was able to regulate GPX4 protein expression, we measured GPX4 protein in the SFRS9-OE transfected Caco-2 cells or SFRS9 shRNAs transfected HCT116 cells. As shown in **Figures 6A, B**, GPX4 protein expression was clearly elevated upon SFRS9-OE in CRC cells, while it was clearly decreased in SFRS9-inhibited CRC cells. Subsequently, RNA immunoprecipitation experiments was carried out in HCT116 cells to confirm that SFRS9 was able to pull-down GPX4 mRNA specifically (**Figure 6C**).

### SFRS9 Affected the Viability and Ferroptosis of CRC Cells Through Regulating GPX4

**Figure 7:**
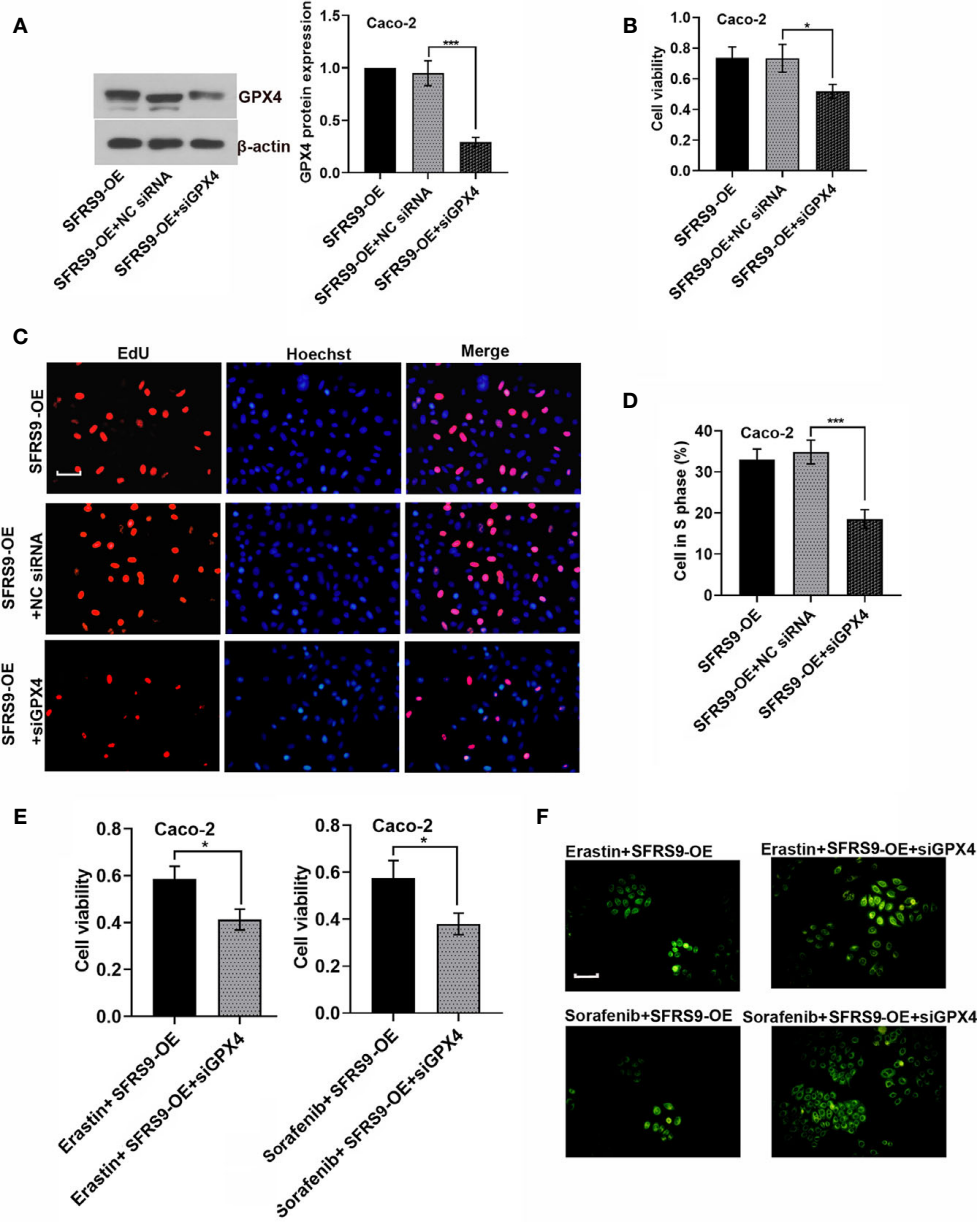
SFRS9 affected the viability and ferroptosis of CRC cells through regulating GPX4. **(A)** The protein levels of GPX4 in Caco-2 cells co-transfected with SFRS9-OE vector and siGPX4. **(B)** The viability of Caco-2 cells co-transfected with SFRS9-OE vector and siGPX4 was measured using the CCK-8 assay. **(C, D)** Representative images and data analysis of EdU staining of Caco-2 cells co-transfected with SFRS9-OE vector and siGPX4. Scale bars: 50 μm. **(E)** The viability of Caco-2 cells co-transfected with SFRS9-OE vector and siGPX4 in presence or absence ferroptosis inducer erastin or sorafenib. **(F)** C11-BODIPY staining of Caco-2 cells following transfection with SFRS9-OE vector and siGPX4 in presence or absence ferroptosis inducer erastin or sorafenib. Scale bars: 100 μm. Values are mean ± SD. ^*^p < 0.05, ^***^p < 0.001.

To confirm that SFRS9 exerts its pro-CRC effects through regulating GPX4, Caco-2 CRC cells were co-transfected with SFRS9-OE vector and siGPX4. SiGPX4 administration significantly inhibited the levels of GPX4 protein in SFRS9-overexpressed cells (**Figure 7A**). Indeed, Caco-2 cell viability and proliferation increased by SFRS9-OE was inhibited by GPX4 knockdown (**Figures 7B–D**). In addition, siGPX4 transfection reversed the inhibitory effects of SFRS9-OE on the erastin/sorafenib-induced ferroptosis, reflecting a decrease in cell viability and an increase in oxidized lipid level (**Figures 7E, F**).

### Effects of SFRS9 on CRC Tumorigenesis *In Vivo*

**Figure 8:**
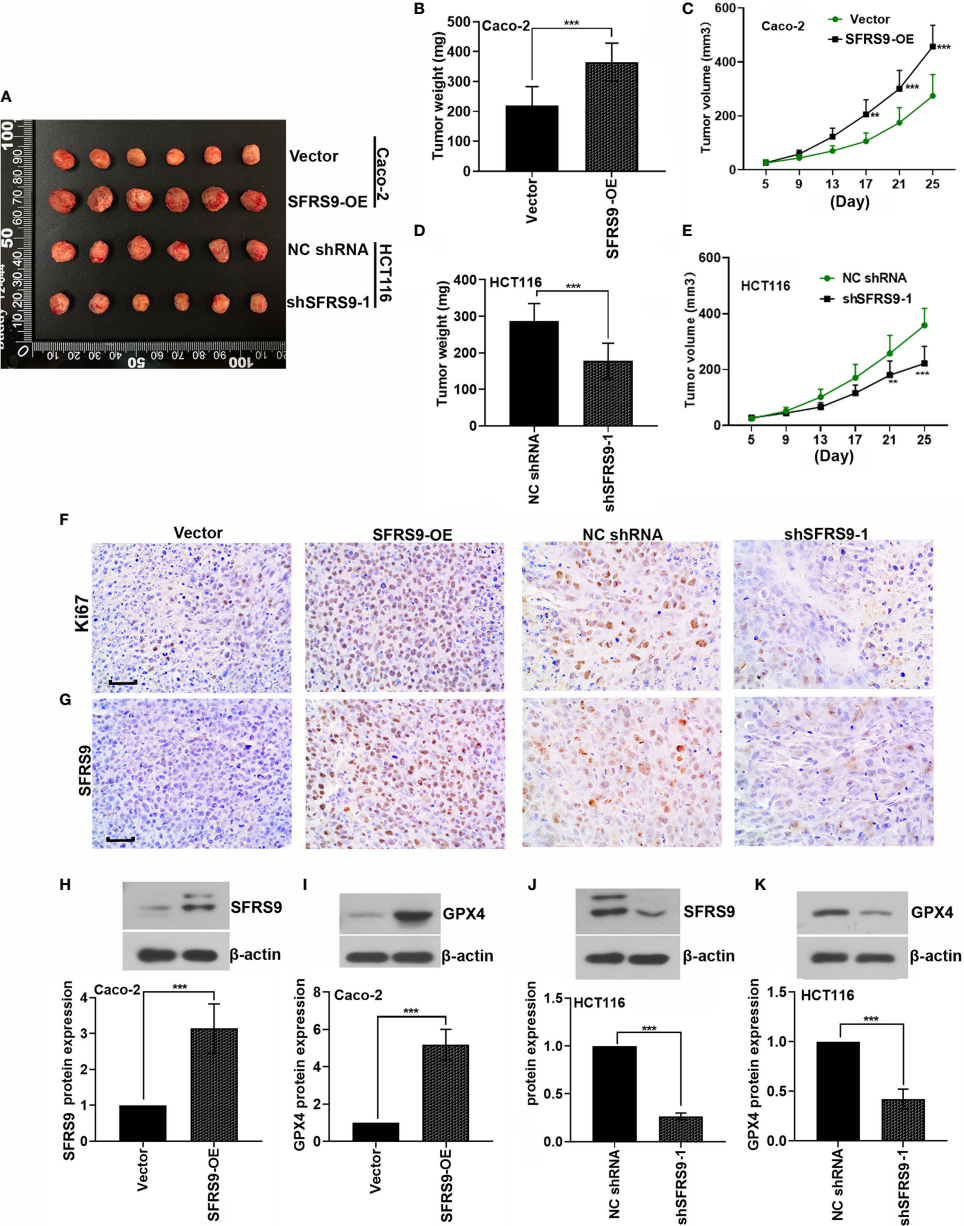
Effect of SFRS9 on the growth of CRC *in vivo*. **(A)** Six primary tumors from mice injected with SFRS9-OE vector stably transfected Caco-2 cells or shSFRS9-1 stably transfected HCT116 cells. **(B, C)** Tumor growth curves and tumor weight of mice injected with Caco-2 cells containing stably transfected SFRS9-OE vector. **(D, E)** Tumor growth curves and tumor weight of mice injected with shSFRS9-1 stably transfected HCT116 cells. **(F)** Ki67 expression in the tumor tissues from mice with different treatmentS was detedcted by immumohistochemical staining. Scale bars: 50 μm. **(G)** SFRS9 expression in the tumor tissues from mice with different treatment was detected by immumohistochemical staining. Scale bars: 50 μm. **(H, J)** western blot analysis of SFRS9 protein expression in the tumor tissues from mice with different treatments. **(I, K)** western blot analysis of GPX4 protein expression in the tumor tissues from mice with different treatments. Values are mean ± SD. ^**^p < 0.01, ^***^p < 0.001.

SFRS9-OE stably transfected Caco-2 cells or sh-SFRS9 stably transfected HCT116 cells were subcutaneously injected into the right flank of nude mice to determine the functional role of SFRS9 in CRC tumorigenesis *in vivo*. Results showed that SFRS9-OE significantly promoted the growth of tumors (**Figures 8A–C**), while SFRS9 knockdown significantly induced tumor growth inhibition (**Figures 8A, D, E**). IHC staining for Ki67 was performed to detect the cell proliferation in tumor tissues, our results showed that overexpressed SFSF9 obviously increased CRC cells proliferation in tumor tissues (**Figure 8F**). Conversely, inhibited SFRS9 significantly suppressed the cell proliferation in tumor tissues from nude mice (**Figure 8F**). In addition, IHC and western blot assays were performed to determine the expression levels of SFRS9 and GPX4 in tumor tissues. As expected, SFRS9 protein expression was obviously upregulated in mice injected with SFRS9-overexpressed Caco-2 cells (**Figures 8G, H**), while it was significantly downregulated in the tumor tissues of mice with shSFRS9-transfected HCT116 cells injection (**Figures 8G, J**). In addition, we found higher GPX4 expression in SFRS9 overexpressed tumor tissues and lower GPX4 expression in SFRS9 inhibited tumor tissues compared with their respective controls (**Figures 8I, K**).

## Discussion

Although the screening and chemoprevention strategies of CRC have reduced the incidence and mortality of CRC in the past decade (17), there is still a need for finding effective treatment targets for CRC. Triggering ferroptosis in cancer cells is a widely reported effective method for inhibiting tumorigenesis. In the present study, we showed that SFRS9 overexpression aggravated, whereas SFRS9 knockdown inhibited the development of CRC both *in vitro* and *in vivo*. SFRS9 limited erastin or sorafenib induced ferroptosis by promoting GPX4 protein expression. These findings reveal a molecular link between SFRS9 and GPX4 in the control of ferroptosis in CRC, and a possible treatment strategy for CRC by inhibiting SFRS9.

SFRS9 plays important roles in constitutive pre-mRNA splicing, mRNA nuclear export, nonsense-mediated mRNA decay and mRNA translation (13). There is ample evidence to indicate that affecting the alternative splicing of tumor suppressors explains some types of inherited and sporadic susceptibility to cancer (18). In addition, it has been reported that SFRS9 is an enhancer of Wnt/β-catenin signaling, and promotes Wnt signaling-mediated tumorigenesis by enhancing β-catenin biosynthesis at the mRNA translational step (14). Another study conducted by Yoshino et al. have shown that SFRS9 mRNA levels were significantly higher in the clinical BC specimens and bladder cancer cell lines than normal control, and SFRS9 knockdown significantly inhibited cell proliferation, migration, and invasion in bladder cancer cells (15). These studies indicate that SFRS9 exerts a pro-carcinogenic role through multiple mechanisms in various cancers. In our study, we found that overexpression of SFRS9 by transfection of SFRS9-OE vector promoted cell viability, proliferation, cycle progression and colony formation. By contrast, SFRS9 silencing significantly inhibited these malignant behaviors of CRC cells. Consistent with previous studies, SFRS9 functioned as a proto-oncogene in CRC cells.

Ferroptosis was first termed by Dixon et al. as a unique iron-dependent form of nonapoptotic cell death (19). Ferroptosis initiator erastin like glutamate, causes the inhibition of the cystine/glutamate antiporter system Xc^−^, thus triggering iron-dependent oxidative death (19). Drug resistance of cancer cells is one of the biggest obstacles in cancer treatment. Ferroptosis as a new form of cell death shows great potentials in cancer treatment particularly for malignancies those are resistant to traditional therapies. Bioinformatics analysis indicated that SFRS9 can bind to GPX4 mRNA. GPX4 is a well-known central regulator of ferroptosis. Thus, we next focused our attention on exploring the effects of SFRS9 on ferroptosis in CRC cells. Our data showed that both ferroptosis inhibitor ferrostatin-1 and apoptosis inhibitor ZVAD-FMK reversed SFRS9 knockdown induced death of CRC cells. Interestingly, ferroptosis inhibitor induced a slight increase in the prevention of SFRS9 knockdown-induced death than the other inhibitor. These data indicated that SFRS9 inhibition could induce CRC cell death through ferroptosis form partly, at least. It has been reported that SFRS9 knockdown by siRNA increased apoptosis of bladder cancer cells (15). Furthermore, in SFRS9 overexpressed cells, ferroptosis inducers erastin or soranefib increased cell viability and lipid peroxidation. Thus, SFRS9 exerted its function in CRC cells was through regulating ferroptotic cancer cell death.

GPX4, a peroxidase enzyme, protects cells against oxidative damage. Direct genetic evidence provided by Friedmann Angeli et al. show that the knockout of GPX4 causes cell death in a pathologically relevant form of ferroptosis (20). GPX4 has been described as an essential regulator of ferroptotic cancer cell death (21). In this study, direct binding between SFRS9 and GPX4 mRNA was experimentally confirmed by RNA immunoprecipitation assay. Reportedly, it is most likely that SR proteins regulate β-catenin at the mRNA translational step (14). Thus, it is possible that SFRS9 can promote GPX4 accumulation *via* binding GPX4 mRNA and enhancing its protein expression. Based on these, we knocked down GPX4 in SFRS9-overexpressed CRC cells and the consequent phenotypes including cell viability and cell proliferation were interrogated. In SFRS9 overexpressed cells, knockdown of GPX4 inhibited the promoting effects of SFRS9 on the cell viability and proliferation. Furthermore, siGPX4 transfection reversed the inhibitory effects of SFRS9-OE on the erastin/sorafenib-induced ferroptosis. These observations indicated that SFRS9 regulated ferroptosis of CRC cells *via* modulation of GPX4. In fact, previous papers have shown that GPX4 inhibition mediated by various small molecules or proteins induced ferroptosis (22, 23). Also, we investigated the role of SFRS9 in CRC tumorigenesis *in vivo*. Consistent with our *in vitro* observations, SFRS9 promoted the growth of tumors and SFRS9 knockdown significantly induced tumor growth inhibition in nude mice, and GPX4 expression in tumor tissues was consistent with that of SFRS9. Collectively, SFRS9 is a pro-oncogene and it inhibits ferroptosis by upregulating GPX4 expression in CRC. Knocking down SFRS9 might be an effective treatment for CRC.

## Conclusion

In summary, SFRS9 represents an obstructive factor to ferroptosis by upregulating GPX4 protein expression, and targeting SFRS9 might be an effective treatment for CRC.

## Data Availability Statement

The original contributions presented in the study are included in the article/**Supplementary Material**. Further inquiries can be directed to the corresponding author.

## Ethics Statement

The studies involving human participants were reviewed and approved by the Ethics Committee of the Shengjing Hospital of China Medical University. The patients/participants provided their written informed consent to participate in this study. The animal study was reviewed and approved by the Ethics Committee of the Shengjing Hospital of China Medical University.

## Author Contributions

RW and ZY contributed to the conception or design of the work, drafted the work or revised it critically for important intellectual content. RW, QS, and HY were involved in the acquisition and analysis of data for the work. RX, DW, and CL interpreted the data for the work. All authors contributed to the article and approved the submitted version.

## Funding

This study was funded by 345 Talent Project of Shengjing Hospital of China Medical University.

## Conflict of Interest

The authors declare that the research was conducted in the absence of any commercial or financial relationships that could be construed as a potential conflict of interest.

## Abbreviations

SRSF9, serine and arginine rich splicing factor 9; CRC, colorectal cancer; OE, overexpression; GPX4, glutathione peroxidase 4; ROS, reactive oxygen species; RRM, RNA recognition motif; IHC, immunohistochemistry; FBS, fetal bovine serum.
