# Supplementary figures and images for: Knockdown of SFRS9 Inhibits Progression of Colorectal Cancer Through Triggering Ferroptosis Mediated by GPX4 Reduction
Source: Front Oncol. 2021 Jul 16;11:683589. doi: 10.3389/fonc.2021.683589 (PMC8322952; doi:10.3389/fonc.2021.683589)

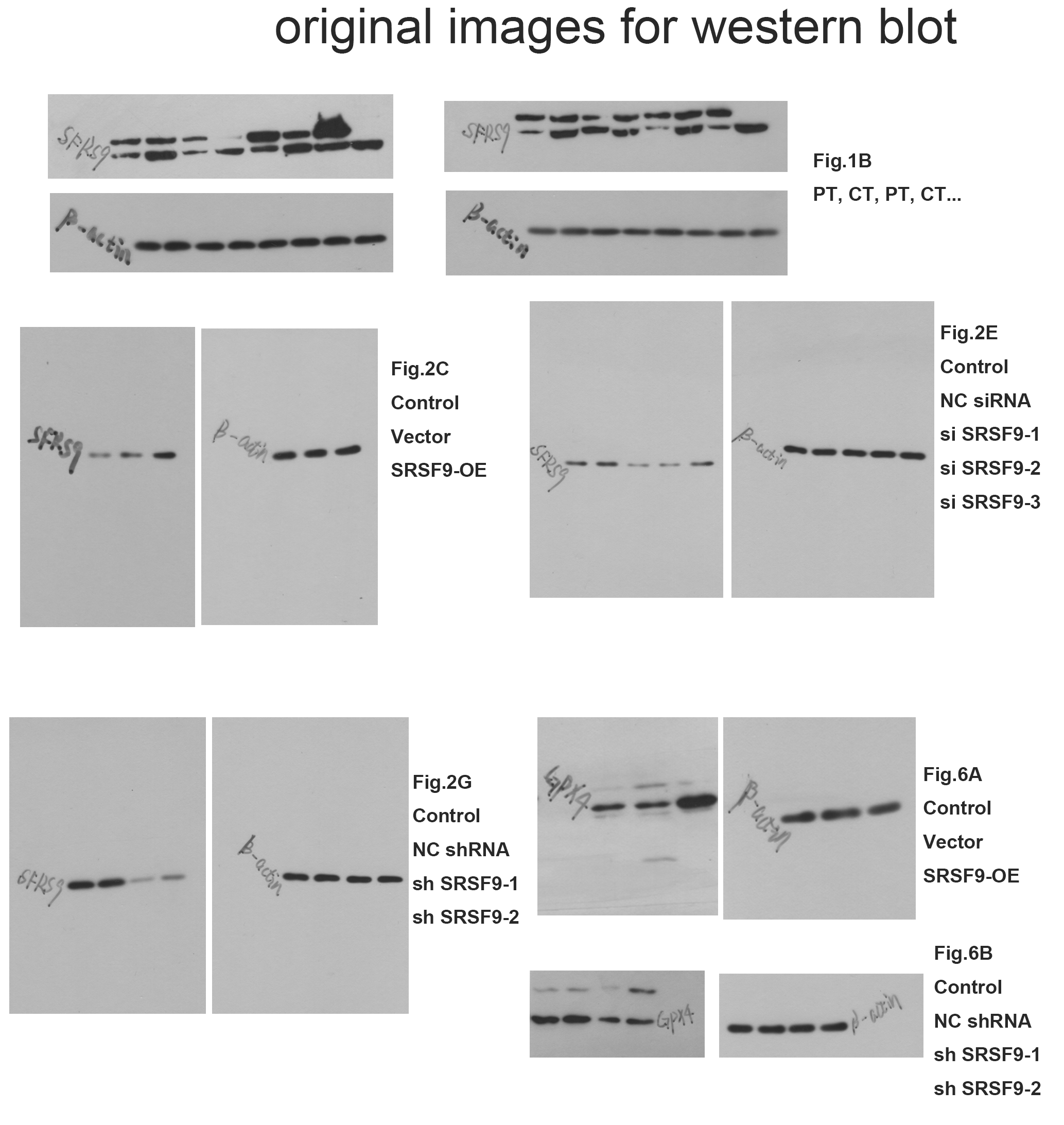

Supplement: Supplementary file 1 [file Image_1.tif]

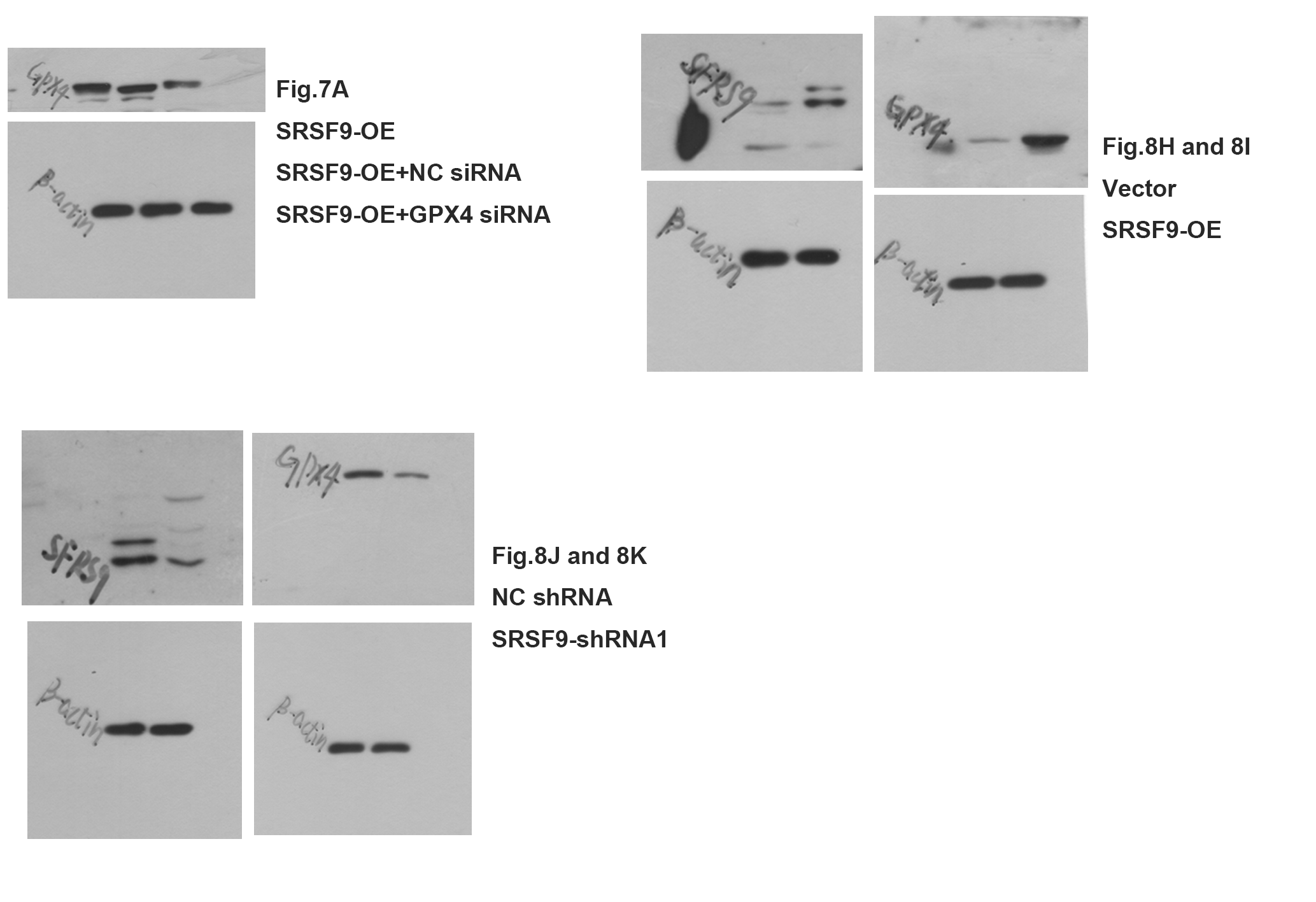

Supplement: Supplementary file 2 [file Image_2.tif]

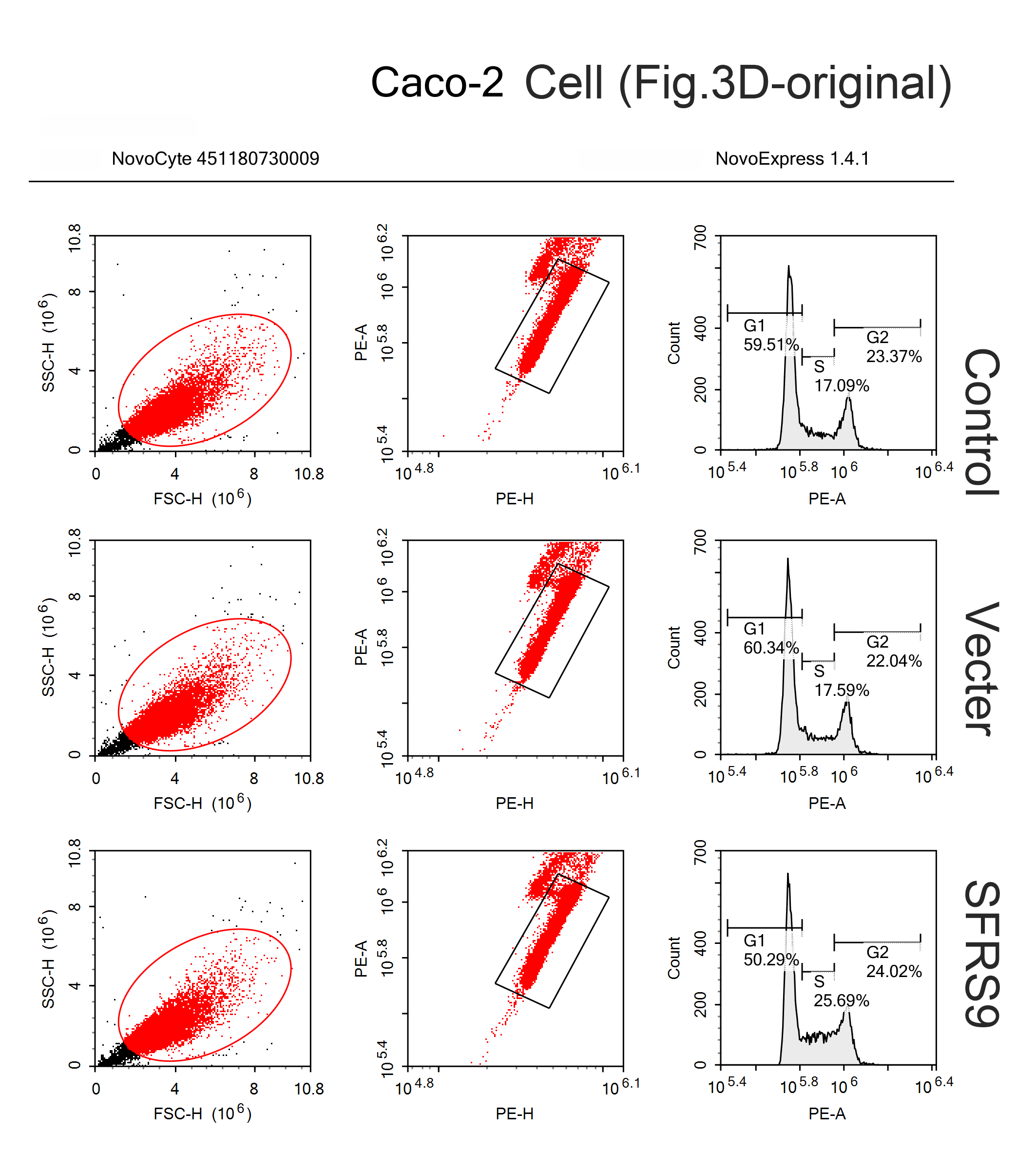

Supplement: Supplementary file 3 [file Image_3.tif]

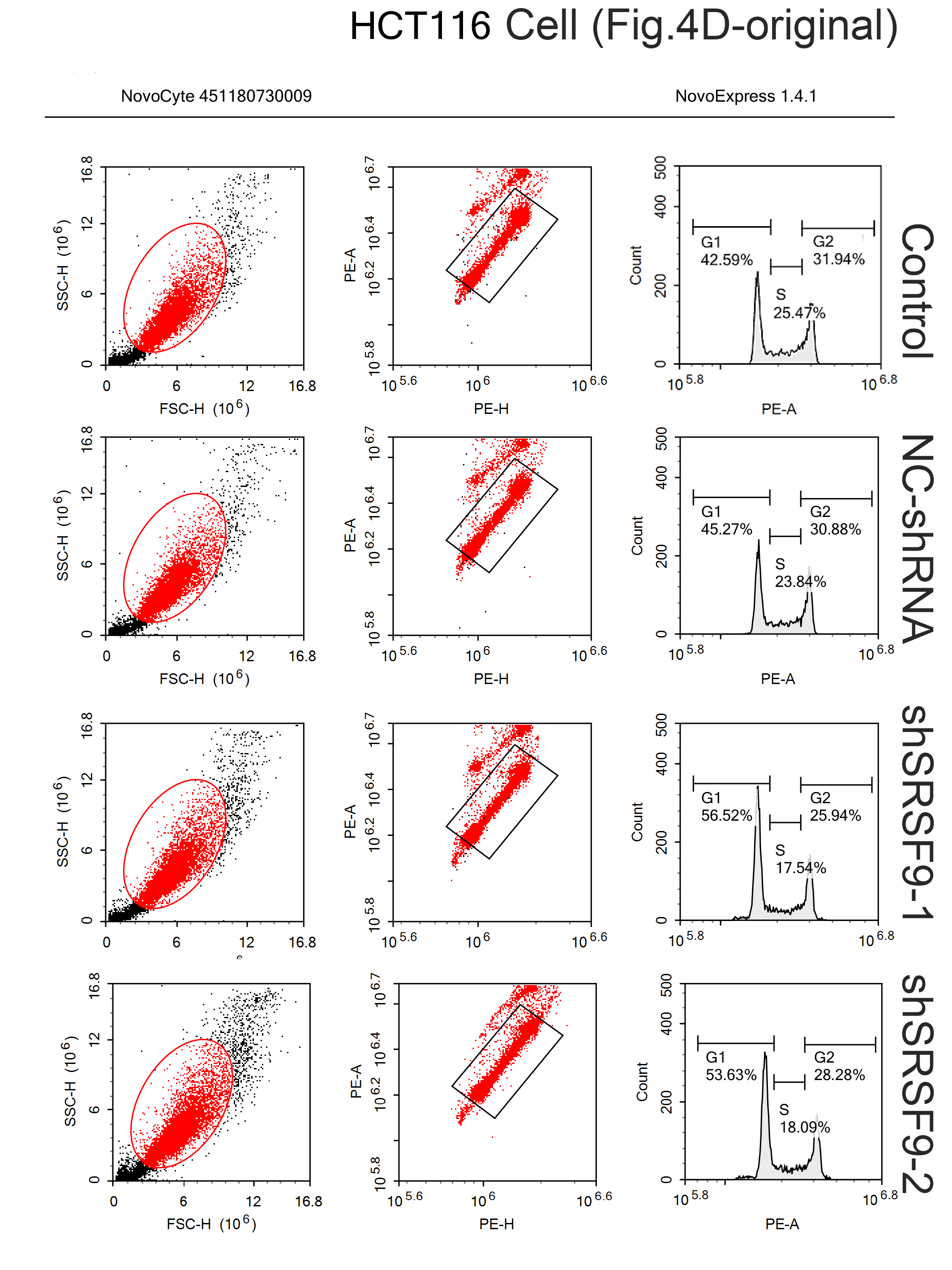

Supplement: Supplementary file 4 [file Image_4.tif]
